# Toward the Improvement of Silicon-Based Composite Electrodes via an In-Situ Si@C-Graphene Composite Synthesis for Li-Ion Battery Applications

**DOI:** 10.3390/ma16062451

**Published:** 2023-03-19

**Authors:** Adrien Mery, Yves Chenavier, Coralie Marcucci, Anass Benayad, John P. Alper, Lionel Dubois, Cédric Haon, Nathalie Herlin Boime, Saïd Sadki, Florence Duclairoir

**Affiliations:** 1 Université Grenoble Alpes, CEA, CNRS, IRIG-SyMMES, F-38000 Grenoble, France; 2 Université Grenoble Alpes, CEA, LITEN, DTNM, F-38054 Grenoble, France; 3 Université Paris Saclay, IRAMIS, UMR NIMBE, CEA Saclay, F-91191 Gif-sur-Yvette, CEDEX, France; 4 Université Grenoble Alpes, CEA, LITEN, DEHT, F-38054 Grenoble, France

**Keywords:** silicon-based composite, Si nanoparticles, carbon-coating, graphene, Li-ion battery, hydrothermal synthesis, hydrogel

## Abstract

Using Si as anode materials for Li-ion batteries remain challenging due to its morphological evolution and SEI modification upon cycling. The present work aims at developing a composite consisting of carbon-coated Si nanoparticles (Si@C NPs) intimately embedded in a three-dimensional (3D) graphene hydrogel (GHG) architecture to stabilize Si inside LiB electrodes. Instead of simply mixing both components, the novelty of the synthesis procedure lies in the in situ hydrothermal process, which was shown to successfully yield graphene oxide reduction, 3D graphene assembly production, and homogeneous distribution of Si@C NPs in the GHG matrix. Electrochemical characterizations in half-cells, on electrodes not containing additional conductive additive, revealed the importance of the protective C shell to achieve high specific capacity (up to 2200 mAh.g^−1^), along with good stability (200 cycles with an average Ceff > 99%). These performances are far superior to that of electrodes made with non-C-coated Si NPs or prepared by mixing both components. These observations highlight the synergetic effects of C shell on Si NPs, and of the single-step in situ preparation that enables the yield of a Si@C-GHG hybrid composite with physicochemical, structural, and morphological properties promoting sample conductivity and Li-ion diffusion pathways.

## 1. Introduction

Moving away from fossil fuels and limiting global climate change are important challenges our society faces today. Storing renewable energy that is intermittent in nature and providing energy to nomadic devices or to electrical vehicles (EV) count among the issues researchers and developers must target to enable a greener and more sustainable future. Li-ion batteries (LIBs) have been major players in the electrochemical energy storage (EES) market since the beginning of the 1990s, particularly in the portable domain [1,2,3]. Today, they play an increasing role in transportation with the development of hybrid and electric vehicles [4,5,6]. However, the current state of commercial batteries’ three key performance metrics (energy density, power density, and lifespan) still leaves significant room for improvement in order to realize the next generation of electric vehicles. These metrics can be improved by developing and optimizing electrode materials and electrolytes, which will drive the development of future LIBs or related technologies (Na ion-based batteries, LiS batteries, Li-air batteries, etc.) [7,8,9].

In line with these challenges, the development of new active materials to replace traditional ones, such as graphite, is a focal point in the recent literature. Among potential negative electrode materials, silicon (Si) appears to be a very attractive candidate for high energy density LIBs mainly due to its high theoretical specific capacity (3579 mAh.g^−1^ for Li_15_Si_4_ alloy) [10,11]. However, Si-based electrodes suffer from several drawbacks which must be overcome to be a viable substitution for commercially used negative electrode materials like graphite (372 mAh.g^−1^). Indeed, the volume expansion (more than 300%) experienced by silicon during the alloy formation with lithium leads to mechanical fracture and pulverization. In addition, the continuous formation of a non-stable SEI (Solid-Electrolyte Interphase) resulting from pulverization leads to the delamination of the electrode material from the current collector and irreversible degradation of the LIB performance [12]. In order to mitigate these drawbacks and improve the stability of Si-based electrodes, several strategies have been developed, such as the nanostructuration of Si particles to limit the pulverization or the synthesis of silicon composites covered by a carbon shell (Si@C) to buffer volume changes and stabilize SEI. Regarding this latter strategy, different synthesis methods have been used to coat Si NPs with a C shell or embed them in a C matrix. These methodologies include CVD, sol-gel synthesis, in situ synthesis, (laser) pyrolysis, carbonization, thermal plasma, or the elaboration of complex structures as the “Yolk-Shell” type [13,14,15,16,17,18]. Current trends also show that these C shells can be N-doped to enhance conductivity and wettability, and this buffer layer concept is also applied to other intercalation compounds displaying high volume change upon cycling [19,20].

The hierarchical structuration of the Si-containing electrode materials becomes an increasingly important property to control in order to promote conductive pathways inside the volume of the electrode and to limit Li^+^ diffusion lengths. This material architecturation combined with a Si NPs buffered surface using a C layer synergetically improves the electrode performances. Such evolution in material design tends to trigger ternary or even quaternary materials, including Si NPs as active materials, C as a protective shell, and a three-dimensional (3D) matrix. In some examples, the Si itself is structured as a porous matrix covered with C-shell [21,22,23]; however, most often, another C component is used to obtain such structuration. Graphite itself can be a 3D carbon support [24,25], but also complex systems made of CNT and cellulose-derived fibers [26] or of organic compound-derived carbons [23] or multi-components and multi-scale yolk-shell systems [27].

In this context of new LiB electrode formulation [28], the use of graphene and graphene derivatives in association with silicon inside electrode formulations is under intense study [29,30,31,32,33]. So, graphene has been used as a buffer layer co-formally covering Si NPs [32,33,34] or combined with Si NPs modified chemically with molecular ligands, sometimes calcinated afterward [35,36,37,38,39,40,41]. Graphene sheets have also been added as a conductive additive with a two-dimensional (2D) morphology preserving contact between Si NPs and Si@C NPs [33,36,38,40,42,43]. More recently, the 3D architecturation of the active material has also been applied to materials containing graphene. In some cases, graphene remains the coat on the Si NPs, and the architecturation is brought about by Si templating [44]. However, in other cases, graphene is part of the 3D scaffold of these Si NPs containing materials [38,45,46]. In these cases, different processes involving templating have been developed to obtain 3D architectures combining graphene and Si NPs [47,48]. Other technical processes, such as roll-to-roll methodologies, are also applied to the preparation of these composites [49]. Sometimes, ternary or quaternary systems are proposed to promote further electrical conductivity and shorten Li^+^ diffusion paths [50,51,52,53,54,55,56,57,58]. Also, 3D architectures such as those involving the formation of 3D graphene hydrogel (GHG) without templates are interesting routes [46,58]. Undoubtedly, the interest in graphene materials for Si-based battery applications lies in their high mechanical resilience, electrical conductivity, and the mesostructuration that these assemblies provide. These beneficial properties, in addition to those of Si, could lead to an increase in the cycle life and the rate capability of Si-based electrodes.

Most examples of such structured materials involved 3D graphene assemblies and Si NPs. The interest of the approach developed in this paper is to combine such 3D graphene structures with Si NPs coated with a C layer in order to get the dual components’ beneficial effects for Li^+^ diffusion and storage. Different methodologies exist to obtain patterning or structuration of graphene composites sample [59,60]. Still, based on our knowledge of GHG synthesis, we propose in this work an original and effective way to combine a 3D graphene hydrogel network (GHG) with Si@C core-shell NPs as negative electrode materials for LiBs applications. The benefit of the protective C-shell around the Si NPs is also addressed here, as we tested and compared carbon-coated (Si@C) and non-coated Si nanoparticles. The expected role of GHG is to bring a conductive and protective percolating network to maximize the contact between Si or Si@C NPs and bring mechanical resistance to the stress suffered by silicon during cycling. To the best of our knowledge, the only comparable work combining the dual effect of 3D graphene hydrogel structuration to the protective C layer of Si NPs has been carried out on Si NPs bearing a C-shell obtained from the calcination of polydopamine [61]. In our work, high-capacity retention (2200 mAh.g^−1^ over 200 cycles) was obtained for the Si@C-GHG composite electrode showing its potential use as a silicon-based composite electrode for Li-ion battery applications.

## 2. Materials and Methods

### 2.1. Synthesis of Si@C Core/Shell Nanoparticles by Laser Pyrolysis

The Si and Si@C NPs were prepared using a laser-induced synthesis method, which has already been described before [62]. The salient features of this protocol are summarized hereafter. Si@C nanoparticles (NPs) were synthesized by laser pyrolysis in a two-stage setup composed of two superimposed reactors working under the atmospheric pressure of Argon. The interaction between the laser beam (CO_2_ Laser) and a silane (SiH_4_) gas flow at the first stage leads to the synthesis of silicon nanoparticles. An Ar carrier gas transfers these Si NPs to a second reaction chamber, where they intersect again with a laser beam in the presence of ethylene (C_2_H_4_). The interaction between C_2_H_4_ flow and the laser beam results in the decomposition of C_2_H_4_ and the formation of a carbon shell around the silicon cores. The laser power and focalization in the bottom stage are adjusted to favor a crystalline structure of the silicon cores, while in the second stage, the laser causes ethylene decomposition. For the synthesis of pure Si NPs (i.e., without shell), only the bottom stage is used.

### 2.2. Synthesis of the Si-GHG and Si@C-GHG Composites under Hydrothermal Conditions

Graphene oxide (GO) was initially prepared by a modified Hummers and Offeman’s method described in previous work [63]. In the present study, Si or Si@C nanoparticles were added to a 5 mg/mL solution of graphene oxide with a %weight ratio of 50/50. The mixture was then sonicated in a sonication bath for approximately 15 min until a homogeneous brownish solution was obtained. This mixture was then transferred into a Teflon-lined stainless-steel autoclave and heated at 180 °C for 1 h or 18 h. Finally, after GO hydrothermal reduction and graphene/Si or Si@C NPs assembly formation in situ, the hydrogel composites Si-GHG and Si@C-GHG were obtained and freeze-dried at −37 °C for 48 h.

### 2.3. Structural and Chemical Analysis

The microstructures of the selected materials were first examined by scanning electron microscopy (SEM, Gemini ZEISS, Oberkochen, Germany) at different magnifications with an EHT of 5 kV and 10 kV. NPs samples were also characterized by transmission electron microscopy (TEM) (Philips CM12, 80 kV) and by high-resolution TEM (HRTEM) (Philips CM200, 150 kV) (Philips, Amsterdam, Netherlands).

Raman spectra were acquired for Si@C from the Horiba XploRA PLUS apparatus (Horiba, Kyoto, Japan) with a 532 nm Ar^+^ laser with a laser power of 0.79 mW.cm^−2^ in order to avoid the evolution of samples under illumination.

BET surface measurements were performed using a Micromeritics Automat 23 (Micromeretics, Merignac, France) to determine the Si and Si@C NPs surface area. The S_BET_ was obtained from a single-point measurement and injected in the following equation:

d = 6/(ρ × S_BET_)

where d is the diameter (nm), ρ is the density, and S_BET_ is the NP’s surface area determined by BET.

X-ray diffraction (XRD) with a wide-angle X-ray diffraction system on a Panalytical X’pert PRO X-ray diffractometer (Malvern Panalytical, Malvern, UK) using a Co Kα radiation source (λ = 1.79 Å)—unless otherwise stated—was used to characterize the structure of Si@C nanoparticles. Transmission experiments have been performed on pellet samples of pristine materials.

Small Angle X-rays Scattering (SAXS) measurements were performed in transmission geometry using a home-made SAXS camera utilizing a point source (size ~200 µm × 200 µm) Bruker-Nonius (FR591) rotating anode generator with Cu-Kα radiation (λ = 1.5718 Å^−1^) at 45 kV and 66 mA. A VANTEC-2000 (Bruker, Billerica, MA, USA) gas-filled area detector (surface 14 cm × 14 cm) was placed at a distance of 350 cm to record the SAXS patterns. The distance calibration was performed using silver behenate as a reference sample. The SAXS profiles were obtained by reduction of the two-dimensional data by radial integration of the intensity after data correction for the background intensity from the empty beam. The sample preparation for the analysis consisted of bulk materials analysis in pristine forms (in-situ synthesis of Si or Si@C NPs-GHG).

X-ray photoelectron spectroscopy (XPS) analyses were performed using a PHI Versa Probe II spectrometer (ULVAC PHI, Inc., Chigasak, Kanagawa, Japan) with a monochromatized Al Kα X-ray source (1486.6 eV) focalized to a spot of 100 µm and with an electron take-off angle of λ = 45°. Samples were studied with powder of pristine samples stuck onto a conductive Cu tape piece mounted onto the sample holder. Survey spectra of the photo-emitted electrons were recorded with a pass energy set at 117.4 eV. High-resolution spectra were acquired with a pass energy of 23.5 eV. Spectra have been recorded in different areas. Data treatments have been performed with MultiPack v.9.5.0.8 software. All spectra were calibrated with respect to the graphene C1s signal at 284.3 eV.

The electrical conductivity of the pristine samples compressed in a pellet was also evaluated by four-point probe equipment and software from Signatone (Gilroy, CA, USA). A current is injected from the pair of external probes, and a potential difference is measured between the two internal probes. These measurements were made in different areas of the pellet, and the mean sample resistance Rs was determined and allowed to calculate the resistivity of the sample via the following equation:

rho = Rs × t

where rho is the sample resistivity, Rs is the sample resistance, and t is the thickness of the sample. Pellet making allows leveraging of the contact resistance arising from graphene stacks to graphene stacks inter-particular resistance in the various samples. It is also noteworthy that this bulk analysis does not allow to subtract contact resistance, but from one sample to another, it is possible to get a tendency of bare sample intrinsic resistance. The samples’ conductivities are extrapolated from the inverse of the resistivity.

### 2.4. Electrode Preparation and Electrochemical Characterizations

Si-GHG and Si@C-GHG composite materials (80 wt%) and CMC binder (20 wt%) were mixed together (without other conductive carbon additives) using a mortar and pestle. The slurry contained 10 mg of active material and 2 mg of CMC and isopropyl alcohol (IPA) until a desirable consistency was obtained. The resulting ink is cast onto a copper foil by doctor blade process and dried overnight at 80 °C. Electrodes with a thickness between 25 and 30 µm were obtained. The electrode material loading is around 1.2 mg per electrode (1.54 cm^2^), i.e., 0.8 mg/cm^2^. The final amount of Si active material on the electrode is evaluated using the initial loading in Si or Si@C NPs. All capacity values are calculated with respect to the mass of Si/Si@C NPs active material, which was around 0.5 mg (i.e., 0.33 mg of Si/cm^2^). The electrochemical characterizations were conducted on a potentiostat/galvanostat (VMP3, Biologic, Seyssinet-Pariset, France). Battery tests were performed in half-cells versus a lithium foil as a counter electrode. The battery electrolyte solution is a mixture of 1 M lithium hexafluorophosphate in ethyl carbonate: diethyl carbonate (1:1) with 10% fluoroethylenecarbonate and 2% vinylene carbonate, abbreviated 1 M LiPF_6_ in EC: DEC (1:1) with 10% FEC and 2% VC. The latter additives have already been shown to be important in trying to stabilize the Si surface [64]. In this work, 150 μL (large excess) of this electrolyte was added to a CR2032 coin cell for electrochemical testing.

## 3. Results

**Figure 1:**
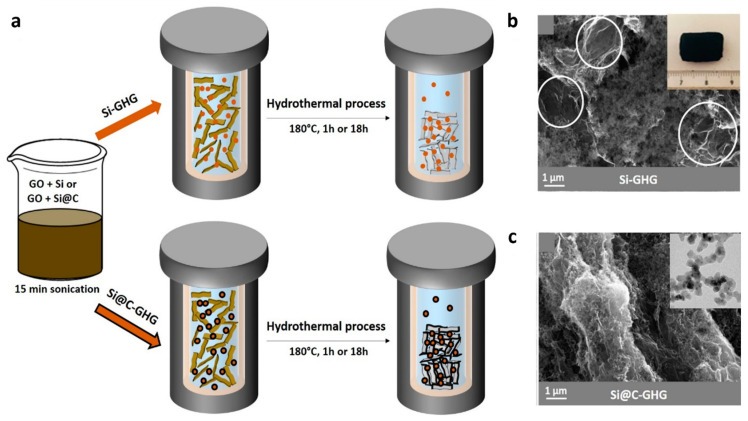
Scheme of the synthesis of Si-GHG (top) and Si@C-GHG (bottom) (**a**); SEM image of Si-GHG composite after 18 h synthesis; within inset a picture of the corresponding macroscale monolith; with white circles showing zones without Si NP (**b**); SEM image of Si@C-GHG composite after 18 h synthesis; within inset a TEM image of the pristine Si@C NPs used in the study (**c**).

The scheme of the hydrothermal process leading to the synthesis of the Si-GHG and Si@C-GHG foams is presented in Figure 1. Si or Si@C NPs are added into an aqueous suspension of GO. The weight percentage of 50/50 NPs/GO has been chosen in order to get sufficient graphene aerogel surface to host the NPs while keeping the amount of Si NPs high enough to achieve high energy densities. Different reaction times have been tested. It was already reported that a 1 h hydrothermal process leads to a less dense graphene hydrogel, while after 18 h, a more compact hydrogel is obtained [65]. Hence, varying reaction times enabled the preparation of samples with differences in their morphologies and porosities of the graphene network. Macroscopically, we also observed that 18 h gels were more compact than 1 h gels. Notably, the Si-GHG composite was prepared to provide a reference enabling the assessment of the impact of the C shell on the electrochemical storage performances. Prior to their electrochemical evaluation, these samples were characterized thoroughly in order to establish a link between their main physico-chemical/morphological properties and their storage performances. The as-obtained Si/graphene composites are labeled Si-GHG-1h and Si-GHG-18h for a synthesis duration of 1 h and 18 h, respectively. Following the same nomenclature, Si@C/graphene composites are labeled Si@C-GHG-1h and Si@C-GHG-18h.

**Figure 2:**
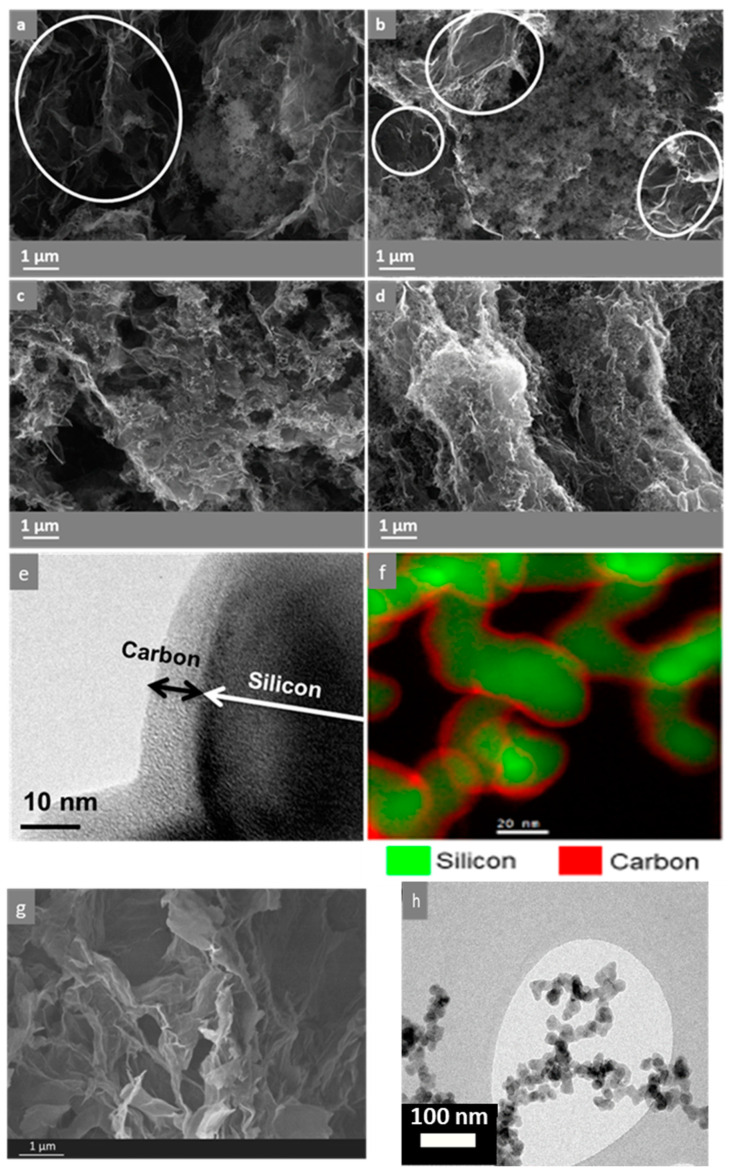
SEM images of Si-GHG-1h (**a**), Si-GHG-18h (**b**) (with white circles showing areas with low amount of SiNPs), Si@C-GHG-1h (**c**), Si@C-GHG-18h (**d**). TEM image (**e**) and STEM-EELS (**f**) images of Si@C NPs highlight the carbon shell around Si NPs. SEM images of reference GHG sample (not filled with Si nor Si@C particles) synthesized in 18 h (**g**). TEM image of Si@C NPs (**h**).

TEM images of both Si and Si@C NPs (Figure 2h and Figure S1) reveal a morphology arranged in a chain-like structure, typical of gas phase synthesis. Both samples exhibit rather similar NPs mean sizes of 22.1 ± 6.8 and 23.3 ± 5.4 nm for Si and Si@C, respectively, with narrow size distributions (Figure S2, counts on 100 particles), consistent with the same Si core but with a carbon shell in the Si@C case. These sizes are also consistent with diameters of 28.7 nm and 29.2 nm, respectively, as determined by BET measurements. The difference between the BET method and TEM average sizes is related to the agglomeration of particles formed by this method, which decreases the surface area (i.e., increases the diameter as calculated from BET measurements).

The carbon percentage in the Si@C sample—determined by ICP—is 14 wt%. From this carbon content, its thickness can be estimated at around 5–10 nm, in agreement with typical TEM and STEM-EELS pictures (Figure 2e,f). The nature of the C shell of the Si@C NPs was also investigated by Raman analysis [66]. The fitted spectrum with D and G bands of carbon is presented in Figure S3. From the deconvolution, it is possible to calculate the R2 ratio (D1/[G + D1 + D2] area ratio) to evaluate the degree of carbon organization [67]. For the Si@C NPs, R2 = 0.55. This result indicates that the carbon is organized but not very graphitic. Indeed, Beyssac et al. indicate that the smaller the index R2, the more organized the carbon is. The values obtained for graphite are lower than 0.2, for example.

**Figure 3:**
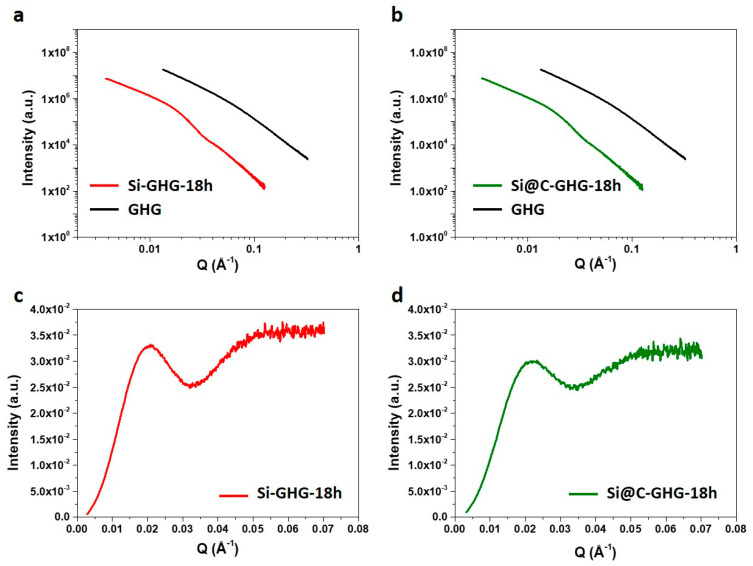
SAXS profiles log(I) vs. lo(Q) for (**a**) Si-GHG and (**b**) Si@C-GHG composite samples obtained by in-situ hydrothermal synthesis samples compared with the SAXS profile for the reference GHG graphene hydrogel. An insight into the scattering in the high Q range is provided by I(Q)^4^ vs. Q plots for both Si-GHG (**c**) and Si@C-GHG (**d**) samples that highlight the contribution from individual Si and Si@C nanoparticles to the overall scattering.

Moving now to composite gel characterization, the sample’s structure was investigated using SAXS to get more insights into the multiscale morphology and porosity of the samples. The scattered intensity profiles I(Q) recorded for pristine GHG, Si-GHG, and Si@C-GHG obtained after 18 h of reaction are shown in Figure 3. A Q^−2^ behavior is clearly visible at low angles in the Q range < 0.02 Å^−1^ (Figure 3a,b). Such behavior is also observed on the I(Q) profile recorded for the reference GHG hydrogel sample, as expected for sheet-like materials. Furthermore, although the intensity for the reference GHG sample shows a linear decrease in log(I) vs. log(Q) representation for larger values of Q (in Q range > 0.02 Å^−1^), a marked oscillation can be observed in this Q range for Si-GHG and Si@C-GHG samples. This oscillation is more clearly evidenced in IQ^4^ vs. Q representation (Figure 3c,d) and indicates agreement with the form factor of individual spherical nanoparticles. The IQ^4^ curves present a first minimum at Qm values around 0.0330 Å^−1^ and 0.0345 Å^−1^ for Si-GHG and Si@C-GHG samples, respectively. The nanoparticles’ diameters of 27.3 nm (Si) and 26.1 nm (Si@C) deduced from these Qm values (following the relation QmR~4.5 available for spherical particles) are in accordance with sizes deduced from BET and TEM measurements. Table 1 regroups the characteristic sizes obtained by the three methods (TEM, BET, and SAXS). The SAXS signatures of Si and Si@C-GHG samples are very alike. These SAXS observations indicate that this in situ preparation of both Si-GHG and Si@C-GHG materials preserves graphene sheet morphology and leads to an intimate embedment and dispersion of individual nanoparticles through the whole 3D graphene sheet-like network.

Figure 2a–d shows SEM images of the different in-situ formed composites studied in this work. An SEM image of the pristine GHG is shown in Figure 3g. The two different parameters are the type of nanoparticles (Si or Si@C) and the synthesis process duration (1 h or 18 h). These morphological characterization results showed significant differences between the composites obtained with Si NPs (Figure 1b and Figure 2a,b) and Si@C NPs (Figure 1c and Figure 2c,d). Indeed, Si-GHG SEM images show large areas with considerable amounts of Si NPs and smaller ones with bare or scarcely covered graphene sheets with Si NPs (Figure 1b and Figure 2a,b—circles in white). The Si NPs are agglomerated and appear poorly dispersed in the graphene framework. Si@C NPs seem well distributed in the bulk of Si@C-GHG, as graphene sheet assemblies are visible across the images and display a rugosity brought about by the homogeneously distributed Si@C NPs. This disparity in NPs distribution from one composite to the other is visible for both reaction times. These observations suggest a better affinity between graphene sheets and Si@C NPs. This may result from the specific hydrophobic-hydrophobic interaction which occurs between the C shell of the Si@C NPs and the newly hydrothermally reduced graphene oxide. In the case of Si-GHG, the surface of the Si NPs is oxidized as soon as it is exposed to air (and under synthesis conditions), leading to an oxidized Si surface that is hydrophilic and polar. Additionally, the XPS data discussed below indicates that the amount of surface oxidation increases during the hydrothermal reduction of the graphene oxide. While the graphene oxide surface may initially have a favorable interaction with the oxidized surface of the SiNPs, as it is reduced during hydrothermal synthesis, the surface becomes more hydrophobic. Hence the interaction between these components during the hydrothermal reduction process becomes less favorable than their interactions between themselves, leading to segregation. In the case of Si-GHG composite, it is postulated that the presence of this oxidized surface plays a role in the kinetics of reduction of graphene oxide. This point will be further discussed in the XRD and XPS sections. These images (Figure 2) also demonstrate that different reaction times for the same composite do not lead to obvious morphological modifications at this observation scale.

**Figure 4:**
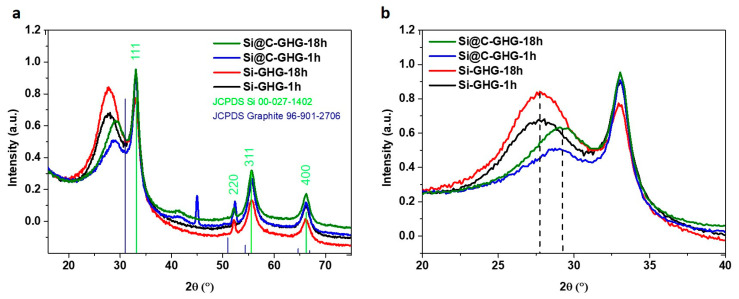
XRD diffractograms of Si-GHG-1h, Si-GHG-18h, Si@C-GHG-1h, and Si@C-GHG-18h (**a**) with a zoom on the graphitic peaks (**b**).

A more local scale (nm range) was probed by performing XRD measurements on the different samples. Results are presented in Figure 4, and complementary XRD information on GHG, Si, and Si@C references are also given in the Supporting Information (Figure S4). The classical signal of silicon material is observed between 33° and 83°. The peaks centered at 33°, 52°, 56.5°, 66°, and 83° correspond to (111), (220), (311), (400), and (331) planes of the diamond cubic crystal structure of silicon, respectively. These observations confirm that these NPs are crystalline. Scherrer analyses of XRD diagrams recorded on Si and Si@C NPs showed that crystalline domains of 6.8 and 6.7 nm, respectively, were visible. As expected, these crystalline domain sizes are smaller than that of the NPs. A graphitic peak (d002) in the area of 28–29.5° was observed for all samples, as expected for such reduced graphene oxide samples, which always show some extent of restacking. Surprisingly, this peak was shifted towards lower angles in Si-GHG composites (2θ~28°) compared to Si@C-GHC composites (2θ~29.5°), whatever the reaction time. The related inter-graphene sheets distance, d, in these RGO stacks (d spacing in XRD), is therefore strongly dependent on whether Si or Si@C NPs had been used to prepare the composite (Figure 4b). However, d values (0.37 nm and 0.35 nm for Si-GHG and Si@C-GHG, respectively) remain in the scale of disordered graphene sheets restacking and do not correspond to graphene sheets separated by NPs. Rather, the larger dSi-GHG compared to dSi@C-GHG implies that graphene oxide in the stacks of Si-GHG composite was less reduced than in Si@C-GHC. An explanation for such phenomena is explained in the XPS section. In turn, the reaction time did not seem to have any effect on the d002 diffraction peaks on the XRD plots recorded for each composite (Figure 4b). The reduction extent seems to reach a plateau after 1 h, as longer reaction time does not lead to evident structural modifications of RGO stacks (that would lead to smaller d) for both Si and Si@C-GHG composites.

**Figure 5:**
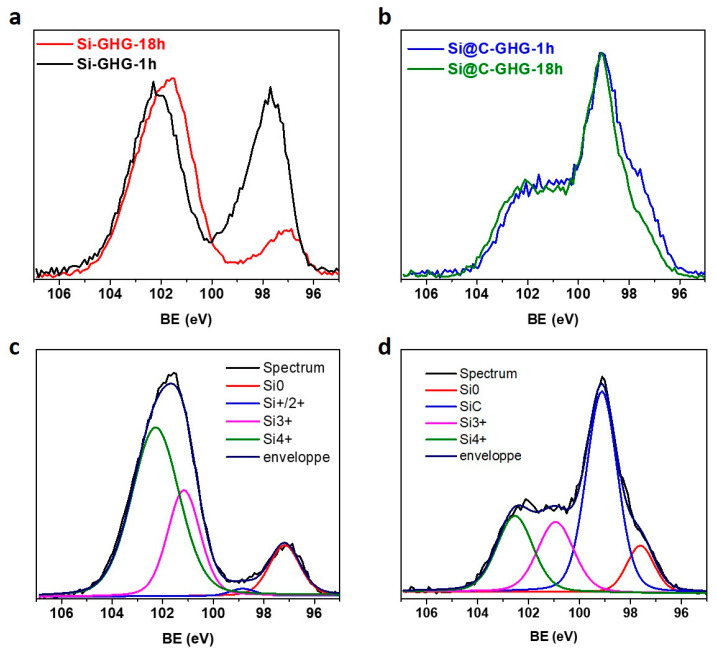
XPS spectra of the four samples. XPS Si 2p HR spectra for Si-GHG-1h and Si-GHG-18h (**a**) and XPS Si_2p_ HR spectra for Si-GHG-18h and Si@C-GHG-18h (**b**). Deconvolutions of the HR Si 2p XPS spectra were recorded for Si-GHG (**c**) and Si@C-GHG (**d**) after 18 h of reaction.

Moving on from morphological and structural characterization, we observed by XPS that reaction time has a strong impact on the Si NPs surface chemistry. First, it is noteworthy that the initial Si 2p HR XPS signal of Si and Si@C NPs shows mainly Si^0^ with slightly less surface oxidation in the presence of the C shell (Figure S5). In more detail, Si 2p HR XPS spectra, recorded on Si-GHG after 1 h and 18 h of the relatively harsh hydrothermal process, showed a very strong increase of the oxidized Si 2p (Si^n+^) peak (102.3 eV) and a decrease of the Si^0^ peak (97.7 eV for 1 h and 97.1 eV for 18 h), indicating nearly complete oxidation of the Si NPs probed surface and certainly deep in the core after 18 h of reaction (Figure 5a) [68]. After 1 h, there are two Si^0^ and SiO_2_ contributions that can be seen as two neat phases in the thin layer model, indicating a core of Si^0^ surrounded by a shell of SiO_2_. When increasing reaction time, the probed volume is comprised completely of ripened SiO_2_ layer, in agreement with the formation of a dielectric native oxide layer. In addition to the Si^0^ peak (97.5 eV), Si 2p HR XPS spectra analyses of Si@C NPs-GHG after 1 h of reaction showed the appearance of a Si^n+^ peak (102.3 eV), indicating partial oxidation of the Si NPs surface (Figure 5b-blue). The peak broadness indicates the co-existence of a diversity of SiOx, SiOx(-C) bonds that can be analyzed as different oxide phases in the probed volume consisting of Si at the interface with the C shell and a portion of bulk/core Si. Following these observations, HR Si_2p_ spectra of the four composites have been deconvoluted using Si^0^, Si^+^, Si^2/3+^, and Si^4+^ components along with a SiC contribution for the Si@C NPs containing composites (Figure S6a,b for both Si-GHG and Si@C-GHG 1 h samples, and Figure 5c,d for both Si-GHG and Si@C-GHG 18 h samples) [68]. The corresponding peak positions and atomic concentrations are reported in Table S1. For the Si@C-GHG composites, the ratio of the atomic percentage of Si 2p and C 1s corresponding to SiC is close to 1 for both reaction times, indicating that these peaks attribution is comprehensive and that this layer is not degraded after 18 h of reaction. For the Si@C composites (Figure 5b-green), only a 1.5× decrease of the ratio of Si^0^/Si^n+^ is observed upon increasing reaction time (0.38 and 0.25 for 1 h and 18 h reactions, respectively), whereas this ratio drastically decreases by a factor of four for the Si-GHG composites (0.51 and 0.13 for 1 h and 18 h reactions, respectively). These observations indicate that the C shell greatly limits the Si core oxidation when using Si@C NPs. However, this shell does not fully shield the core Si, and the evolution of this signal occurs mainly during the first hour of the reaction, as shown by the Si^n+^ signal of the Si@C NPs containing composite (Figure 5b). Such observation can be explained by a modification of the C shell/core Si NPs interface. The C shell is not continuous, as evidenced by STEM-EELS, which allows limited oxidation to occur at this interface. After some time (in the 1 h range), gradient oxidation occurs on this limited interfacial Si surface exposed to air, and no further modification of this layer is observed (even after 18 h). Interestingly, all XPS spectra have been calibrated on the Csp2 signal of graphene at 284.3 eV, leading to low Si^0^ peak positions (97.1–97.7 eV) compared to more standard 98.8 eV [68]. This shift to lower binding energy can arise from the presence of a dielectric SiO_2_ layer leading to a differential charging effect. In agreement with the protective nature of the C layer, this shift is more pronounced for the composites containing bare Si NPs, and increases even further after 18 h (Si^0^ for Si-GHG-1h at 97.7 eV and Si^0^ for Si-GHG-18h at 97.1 eV). These XPS observations show that Si NPs in Si-GHG get covered with SiO_2_ layers that grow thicker with reaction time, while Si@C NPs in Si@C-GHG experience only limited oxidation. In turn, XRD analysis confirmed graphene oxide reduction with variable extent between Si-GHG and Si@C-GHG composites. So, these characterizations show that two processes occur simultaneously during hydrothermal synthesis. Both processes appear to be interdependent, as the oxidation behavior of Si and Si@C NPs seems to impact the kinetics and extent of the concomitant graphene oxide reduction reaction. Such competition between oxidation and reduction reactions leads to this XRD observation of a lower reduction extent of graphene in Si-GHG than Si@C-GHG (observed from dSi@C-GHG < dSi-GHG). Oxidized NPs and graphene oxide must form a more stable suspension in water, not favoring GO reduction, while the introduction of a C layer around the Si NPs must destabilize this initial suspension and, on the contrary, promote the interactions between the C shell and newly reduced graphene, probably displacing the equilibrium towards further graphene oxide reduction.

Electrical conductivity measurements were performed on all samples. The recorded values can also be linked to NPs oxidation (and to some extent to the less complete graphene reduction as evidenced by higher d-spacing and lower XPS C/O ratios for Si NPs-containing samples), as electrical conductivity values of all the Si@C-GHG samples are higher than that of the Si-GHG composites (Table 2), certainly explained by the presence of a high amount of SiO_2_ layers blocking conduction paths. Moreover, it is interesting to note that pycnometer measurements were conducted, and densities of 0.04 and 0.11 g/L were obtained for Si-GHG-18h and Si@C-GHG-18h, respectively. This higher density for Si@C-GHG composite, possibly arising from closer proximity between graphene sheets and Si@C NPs (as evidenced in XRD), could partly explain this higher conductivity. Among Si@C-GHG samples, the highest electrical conductivity was obtained when an 18 h reaction was performed with a value of 696 S/m.

Thus, these latter results confirm a strong impact of the 18 h reaction time on the Si@C-GHG synthesis, which lead to a denser network resulting in higher electrical conductivities. This may suggest an impact on future electrochemical properties.

**Figure 6:**
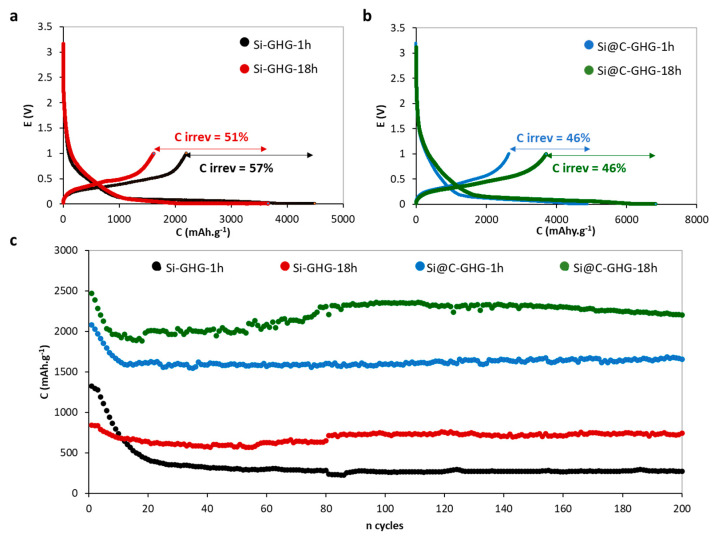
Battery performances of the samples in half-cells in 1 M LiPF6 in EC: DEC (1:1) with 10% FEC and 2% VC electrolyte. First cycles at C/20 with the corresponding irreversible capacities for Si-GHG samples (**a**) and Si@C-GHG samples (**b**). Cycling performances (discharge capacities) of the different samples at C/5 charging/discharging rate (**c**).

After the physicochemical characterizations, electrochemical tests were conducted on the different samples to identify their performance as Li-ion anode materials. The first lithiation/delithiation galvanostatic curves are displayed in Figure 6a,b. A low charge/discharge rate of C/20 is applied during this first cycle to allow a complete and deep lithiation of the composite electrode. This first experiment already showed an important amount of loss of Li ions during SEI formation. Hence, high irreversible capacities (C_irrev_) are recorded (45–60%). Still, it is observed that lower C_irrev_ values were achieved with Si@C-GHG (~46%) compared to that obtained with Si-GHG (~51–57%), indicating that C shell plays a partial stabilizing role in the SEI formation process. Coulombic efficiencies have been reported in Figure S7. Cycling tests were performed on the different half-cells at a C rate of C/5 over 200 cycles (Figure 6c). The results of electrochemical performances are given in Table 3. We observed that specific capacities (C_SP_) were very low and clearly below 1000 mAh.g^−1^ from the first cycles (<10 cycles) for the Si-GHG composites, while they exceeded 1600 mAh.g^−1^ with good stability for Si@C-GHG composites. For most samples, an important capacity drop is observed before the 20th cycle, plausibly explained by the formation of an unstable SEI layer accompanied by a structural evolution of the active material. Specifically for the Si@C-GHG-18h composite, an increase in capacity is taking place between the 20th and the 80th cycles. This rise may be explained by a stabilization of the SEI, combined with a less important morphological modification of the materials, leading to better wettability and a core lithiation of the Si@C NPs. Coulombic efficiencies of Si-GHG-18h and Si@C-GHG-18h during cycling are shown in Figure S7. We could also observe that composites obtained after 18 h yielded higher C_SP_ (for Si-GHG-18h: 750 mAh.g^−1^; for Si@C-GHG-18h: 2200 mAh.g^−1^) than composite prepared in 1 h (for Si-GHG-1h: 270 mAh.g^−1^; for Si@C-GHG-1h: 1670 mAh.g^−1^). These trends can be explained by the morphological and physicochemical properties of both composites. The main differences between samples remain their electrical conductivity and their porosity. For 18 h as reaction time, Si@C-GHG composites are more conductive and denser (Table 2) than the Si-GHG composites. The 18 h reactions also lead to higher conductivity compared to 1 h reactions. These results tend to indicate that the higher the bare sample’s electrical conductivity, the higher the capacity. Highly oxidized Si NPs and hence low conductivity Si-GHG composites yield lower C_SP_. For Si@C-GHG composites, the difference between Si@C-GHG-1h and Si@C-GHG-18h does not arise from different local structures nor from chemical composition (as evidenced by XPS and XRD) but more from a difference in electrical conductivity that explains an enhanced percolation in the graphene 3D network obtained after 18 h reaction, as suggested before. It was shown in the literature before that after 18 h of reaction time, a denser graphene hydrogel assembly is obtained compared to that obtained after 1 h [65]. Such sample densification may explain the improvement in sample conductivity with increasing reaction time.

**Figure 7:**
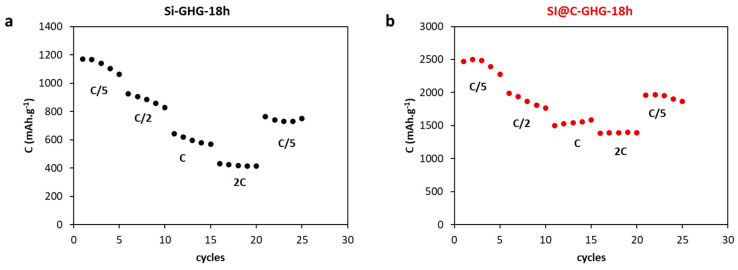
Rate capability tests at several C rates for Si-GHG-18h (**a**) and Si@C-GHG-18h (**b**) samples in half-cells.

Rate capability tests (Figure 7) were conducted on the two best-performing systems with Si NPs (Si-GHG-18h) and Si@C NPs (Si@C-GHG-18h) at different C rates (between C/5 and 2C). Results showed once again enhanced performances for Si@C-GHG composites. The initial capacity for Si@C-GHG-18h is around 2500 mAh.g^−1^ compared to 1200 mAh.g^−1^ for Si-GHG-18h. Upon increasing discharging rate to 2C, the Si-GHG composite retained a 33% capacity, while Si@C-GHG still displayed a 55% capacity retention. On returning to the C/5 rate, both composites lose ~500 mAh.g^−1^ with respect to their initial capacities. Si@C GHG composite recovers a high 1900 mAh.g^−1^ capacity, while the recovered capacity of the Si-GHG composite is only around 700 mAh.g^−1^. In agreement with the previous observations on cycling performances, the specific capacity is greatly enhanced when Si@C nanoparticles are present inside the composites. For the non-protected Si NPs, the harsh hydrothermal process, leading to the in situ formation of the 3D graphene assembly around the NPs, seems to negatively counterbalance the expected benefits of such percolating network in decreasing the composite electrical conductivity. As mentioned before, this effect can be attributed to the large oxidation of Si NPs when non-carbon coated. When Si@C NPs are used, the C shell protects the Si surface, and the 3D percolation network formed with graphene sheets is allowed to reach much higher conductivities as well as higher density, and hence higher C_SP_ and better cyclability.

**Figure 8:**
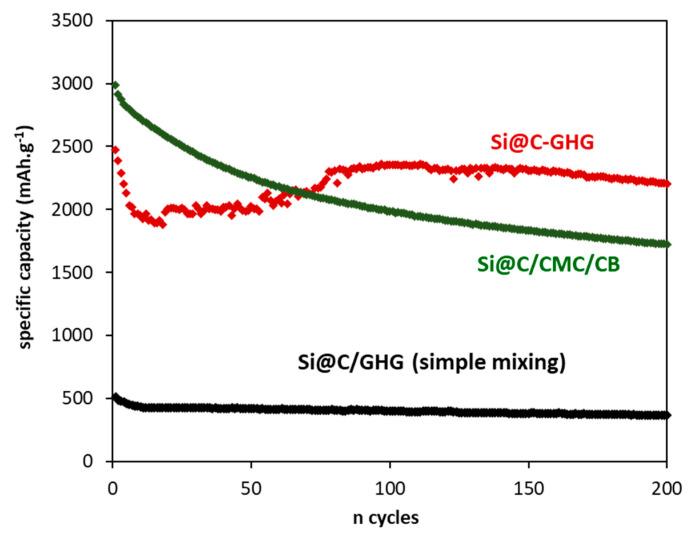
Cycling performances in half-cells of Si@C-GHG (red) after 18 h reaction at a charge/discharge rate of C/5 and comparison with the simple mixing of Si@C with GHG (black) and a standard formulation (green) consisting in a mixing of Si@C NPs, carbon black and CMC—50/25/25 %wt.

To further investigate the role of the 3D assembly, other electrode formulations were also tested. Firstly, a standard electrode formulation—consisting of Si@C, conventional carbon black additive, and CMC binder in 50/25/25 mass ratio—was electrochemically tested in the same conditions (Si@C/CMC/CB in Figure 8). While the initial capacity of this standard formulation is higher (3000 mAh.g^−1^ vs. 2500 mAh.g^−1^ for Si@C-GHG), a constant capacity fading occurs during cycling (1700 mAh.g^−1^ vs 2200 mAh.g^−1^ for Si@C-GHG after 200 cycles). These results show that the use of the 3D Si@C-GHG composite leads to better cycling stability. The other tested electrode formulation consists of a simple mixture of GHG and Si@C NPs (i.e., no in situ formation of GHG around Si@C NPs). For this purpose, the two components were hand mixed in a mortar in the presence of CMC for 20 min. The C_SP_ achieved for this material reached only 500 mAh.g^−1^ (Figure 8 black curve) and maintained a C_SP_ around 400 mAh.g^−1^ after 200 cycles. This capacity is more than 5 times lower compared to the CSP achieved with Si@C-GHG composite. These results clearly highlight the benefits of the 3D in-situ synthesis of Si@C-GHG composite, which surpass the electrochemical performances of the other Si@C formulations and those based on bare Si NPs (Figure 6c). However, the first irreversible capacity, along with the drop in capacity over the first cycles (Figure 6)—likely arising from the high macro porosity of these materials—remain issues to be addressed. Nevertheless, it is noteworthy that the Cirrev recorded for the standard electrode formulations is also quite high (~30%). This problem remains a drawback very dependent on nanoparticles and high surfaces and not only on formulations containing graphene.

**Figure 9:**
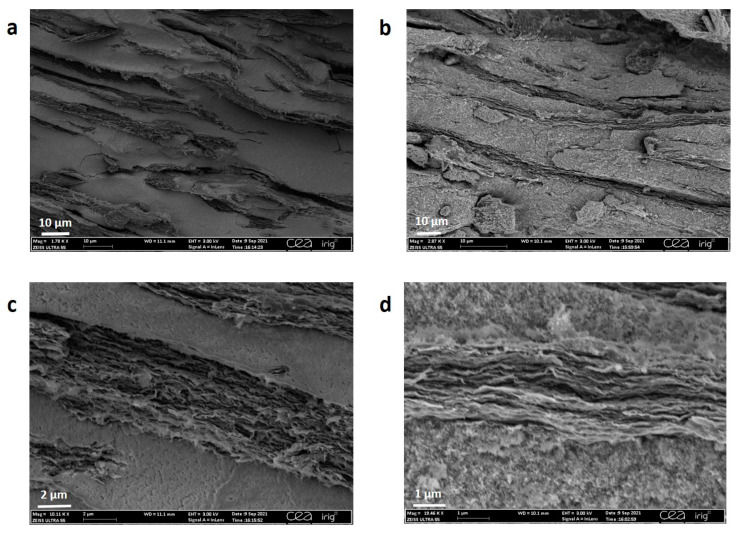
SEM images at different magnifications of (**a**,**c**) Si-GHG and (**b**,**d**) Si@C-GHG samples were obtained by a simple mixture of components.

This decrease in macroscopic electrochemical performances could explain the morphology differences between in situ formed Si or Si@C-GHG composites and simple mixtures of components. Indeed, SEM images (Figure 9) recorded on the mixtures show distinctly that different domains co-exist. Graphene sheets and Si/Si@C nanoparticles are inhomogeneously dispersed. This observation contrasts sharply with the graphene sheets, and Si/Si@C nanoparticles entanglement from the in situ formed Si-GHG and Si@C-GHG samples (Figure 2). Hence, such phase segregation phenomena could explain a loss of electronic contact between Si active material and graphene conductive additive lowering the capacity of the system. In contrast, this electronic contact is preserved in composites with homogeneously distributed samples, explaining the highest capacity values of in situ formed samples.

Altogether, these results underline the interest in using 3D percolating assemblies of graphene sheets incorporating efficiently dispersed Si@C NPs. In mixtures, the efficient interconnection of these two components is not optimum. The in situ preparation involving C shell-protected Si NPs, promoting their homogeneous dispersion, allows us to obtain a hybrid composite synergistically combining the properties of each individual component to yield a material with high Li storage performances. As compared to previously reported state-of-the-art results (presented in Table S2) obtained from electrode materials containing 2D graphene additives or 3D graphene networks as a composite component, the high capacity achieved with Si@C-GHG-18h after 200 cycles (for a formulation not containing additional conductive C additive like Super P) highlights further the interest of this 3D composite and its preparation process for the development of efficient Si-containing active materials for LiBs.

## 4. Conclusions

Graphene hydrogel (GHG) was used as a scaffold for Si@C NPs in order to promote Si active material cycling in Li-ion batteries. Such hybrid materials were prepared by a single in situ hydrothermal process. Three-dimensional composites with Si@C NPs homogeneously dispersed in the graphene hydrogel framework have been obtained. These hybrid composites showed remarkable electrochemical performances with a high capacity of around 2200 mAh.g^−1^, stable over 200 cycles. It also has to be stressed that such value is obtained for an electrode material not containing any additional conducting additive. The positive impact of the carbon shell around Si NPs was established by comparing these results to in-situ formed composites incorporating bare Si NPs in the graphene structure. The hydrothermal process is harsh on Si NPs, but Si@C NPs withstand the process nicely, showing that the C shell efficiently prevents the SiNPs’ deep oxidation. Electrical conductivity seems to be a key parameter that has been shown to depend on the surface state of Si (1st hour), as well as 3D network modification (after 18 h reaction). With the enhancement of conductivity and density modulation resulting in charge diffusion length decrease, the good electrochemical results obtained with Si@C-GHG prove the interest of the graphene 3D conductive architecture combined with the Si NPs protective C shell. Altogether the C_SP_ values achieved are really interesting and highlight the potential of these silicon-based composite electrodes for Li-ion battery applications. The interest in combining electrode 3D structuration to Si NPs surface buffering can also be witnessed by recent work on the use of other sheet-like materials like MXenes to play such a structuration role [69]. The still high irreversible capacity at the first cycle remains an issue to mitigate. Post-mortem/operando analysis could bring further understanding of the mechanisms at stake and orientate towards electrode materials optimization paths. Technological solutions such as prelithiation processes or graphene/Si NPs surface passivation with artificial SEI could be avenues to follow. 

## Figures and Tables

**Table 1:** Comparison of the various Si NPs and Si@C NPs diameters determined by either TEM, BET, or SAXS analyses.

	Characteristic Size (nm)		
	TEM	BET	SAXS
Si NPs	22.1	28.7	27.3
Si@C NPs	23.3	29.2	26.5

**Table 2:** Inter-graphene sheets distance—d—determined from XRD, C/O ratios calculated from XPS atomic concentration data, and electrical conductivities recorded for Si-GHG 1 h and 18 h compared to that of Si@C-GHG 1 h and 18 h. Densities of Si-GHG-18h and Si@C-GHG-18h are reminded.

Samples	d (nm)	C/O	σ (S/m)	ρ (g/L)
Si-GHG-1h	0.37	1.7	232	-
Si-GHG-18h	0.37	1.8	308	0.04
Si@C-GHG-1h	0.35	3.1	388	-
Si@C-GHG-18h	0.35	3.0	696	0.11

**Table 3:** Summary of electrochemical characteristics of the tested samples: irreversible capacity after the 1st cycle, capacity after the 200th cycle, and the average of the coulombic efficiency recorded over 200 cycles, with a reminder of the bare sample conductivity.

Samples	C_irrev_ (%) 1st Cycle	C (after 200 Cycles) mAh.g^−1^	Average Efficiency (%) at 200 Cycles	σ (S/m)
Si-GHG-1h	57	273	99.3	232
Si-GHG-18h	51	745	99.1	308
Si@C-GHG-1h	46	1670	99	388
Si@C-GHG-18h	46	2205	99.1	696

## Data Availability

Data is contained within the article or supplementary material.

## Supplementary Materials

The following supporting information can be downloaded at: https://www.mdpi.com/article/10.3390/ma16062451/s1. Figure S1: TEM images of bare Si NPs. Figure S2: Particle size distributions of Si NPs (a) and Si@C NPs (b) determined from TEM images. Figure S3: Raman spectra of Si@C NPs. These deconvolutions highlight the low degree of organisation of the C-shell. The detailed explanation of the various components and associated interpretations can be found elsewhere [66,67]. Figure S4: complementary XRD diffractogram of the sample references: Si, Si@C (Cu Kα radiation source) and GHG (Co Kα radiation source). Figure S5: Si2p HR XPS spectra of pristine Si (a) and Si@C NP samples (b), showing the low intensity of the SiO_2_ contribution. Figure S6: Deconvolutions of the HR Si2p XPS spectra recorded for Si-GHG (a) and Si@C-GHG (b) after 1h of reaction. Figure S7: Coulombic efficiency of Si-GHG-18h (a) and Si@C-GHG-18h (b) during cycling. Table S1: Recapitulation of the deconvoluted Si2p components (peak position, FWHM values and atomic concentrations) attributed to Si0, SiC, Si+/Si2+, Si3+ and Si4+, along with ratios highlighting the stability of Si@C NPs-GHG composite compared to the more oxidation-sensitive Si-GHG composites (clear decrease of the Si0/SiOx with increasing reaction time). Table S2: Comparison of different electrochemical performances obtained for electrode materials containing Si or Si@C NPs and graphene as an additive (in orange) or as a 3D scaffold (in green) described in the recent literature. It is noteworthy that the frontier between graphene as 2D or 3D component is not always clear-cut.
