# Supplementary material for: Toward the Improvement of Silicon-Based Composite Electrodes via an In-Situ Si@C-Graphene Composite Synthesis for Li-Ion Battery Applications
Source: Materials (Basel). 2023 Mar 19;16(6):2451. doi: 10.3390/ma16062451 (PMC10051277; doi:10.3390/ma16062451)
Supplement: Supplementary file 1 [file materials-16-02451-s001.zip › materials-2234078-supplementary.pdf]

Electronic Supplementary Information

Toward the Improvement of Silicon-Based Composite Electrodes via an In-Situ Si@C-Graphene Composite Synthesis for Li-Ion Battery Applications

Adrien Mery [1], Yves Chenavier [1], Coralie Marcucci [1], Anass Benayad [2], John P. Alper [3], Lionel Dubois [1], Cédric Haon [4], Nathalie Herlin Boime [3], Saïd Sadki [1], Florence Duclairoir [1,*]

1. Université Grenoble Alpes, CEA, CNRS, IRIG-SyMMES, F-38000 Grenoble, France
2. Université Grenoble Alpes, CEA, LITEN, DTNM, F-38054 Grenoble, France
3. Université Paris Saclay, IRAMIS, UMR NIMBE, CEA Saclay, F-91191 Gif-sur-Yvette, Cedex, France
4. Université Grenoble Alpes, CEA, LITEN, DEHT, F-38054 Grenoble, France

* Correspondence: florence.duclairoir@cea.fr

1- Material preparation:

Graphene Oxide (GO) Synthesis: GO synthesis was performed by following a modified-Hummers and Offeman's method. Graphite powder (3.1 g) was dispersed in H_2SO_4 (91 ml) under stirring into a 1 L beaker placed in an ice bath. $NaNO_3$ (1.5 g) was then added and the reaction media was stirred vigorously while maintaining the temperature under 2 °C. After 2h, $KMnO_4$ (9 g) was added portion-wise to keep the mixture temperature below 4oC. The reaction media was kept under stirring at such controlled temperature for a further 1h, before it was let to reach room temperature. Then water (100 mL) was added slowly over a period of 20 minutes maintaining the temperature under 95 °C. The beaker was then placed into an oil bath and stirred at 95 °C. After 1.5 h, water (300 mL) was added making sure the temperature does not rise above 85 °C. Stirring was continued for 1.5 h and then the reaction mixture was let to reach room temperature. H_2O_2 (30 mL) was added and the mixture was stirred for 1h. Finally, water (450 mL) was added and kept for stirring overnight.

The mixture was filtered through a P4 fritted Buchner filter (10–15 µm). The solid graphene oxide cake was re-dispersed in 600 mL of water, and the dispersion was homogenized under stirring for 1h and sonication for a further hour. The mixture was then acidified with 100 mL of HCl and left under stirring overnight. Finally, the GO dispersion was filtered and centrifuged repeatedly until the supernatant reached pH 5. GO solution with a concentration of 5 mg/ml was obtained by carefully measuring the weight of 10 ml of lyophilized GO.

2- Samples characterization

All the various caharcaterization methods described and discussed in the main text have been used. In this SI are also presented STEM-EELS images obtained on the bare nanoparticles. The conditions used for this specific analysis are the following ones: STEM-EELS experiments were conducted using a FEI Titan Ultimate microscope equipped with a monochromator and double spherical aberration correctors (Cs) for both the probe forming and the image-forming lenses. The microscope was operating at an accelerating voltage of 200 kV using a 20 mrad convergence semi-angle. STEM images were collected using a high-angle annular dark field detector, where inner and outer semi-angles were 60 and 180 mrad, respectively. EELS map was acquired using a Gatan GIF Quantum in the Dual EELS mode allowing the simultaneous collection of the low loss and core loss spectra. Acquisition times were 1 µs and 20 ms respectively and the collection semiangle was 35 mrad.

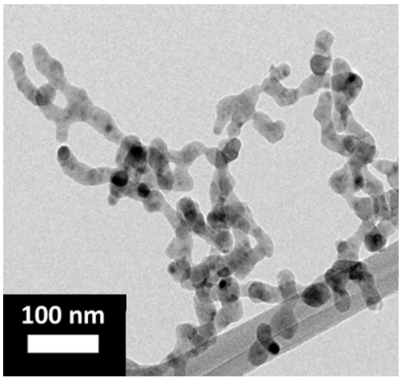

Figure S1: TEM images of bare Si NPs.

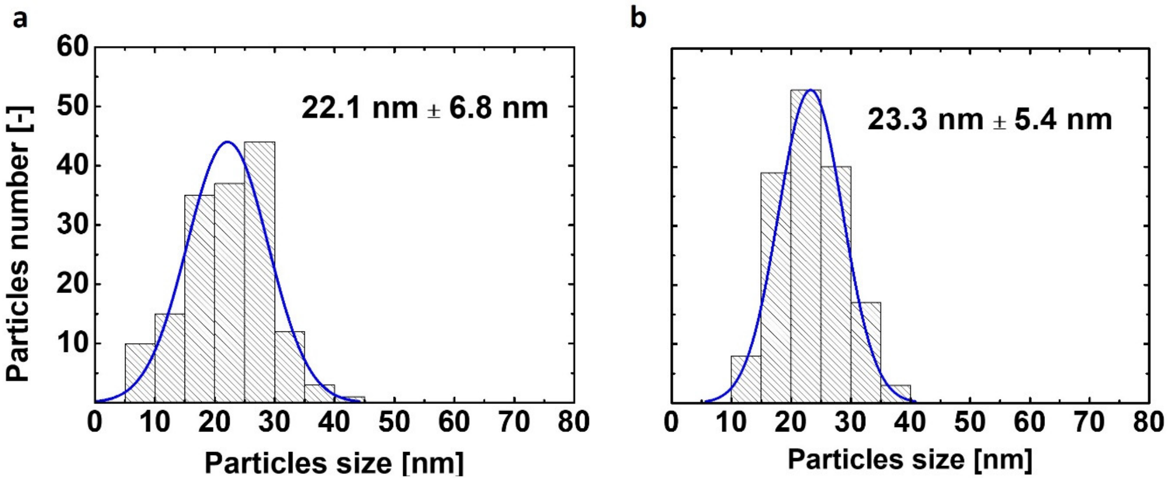

Figure S2: Particle size distributions of Si NPs (**a**) and Si@C NPs (**b**) determined from TEM images.

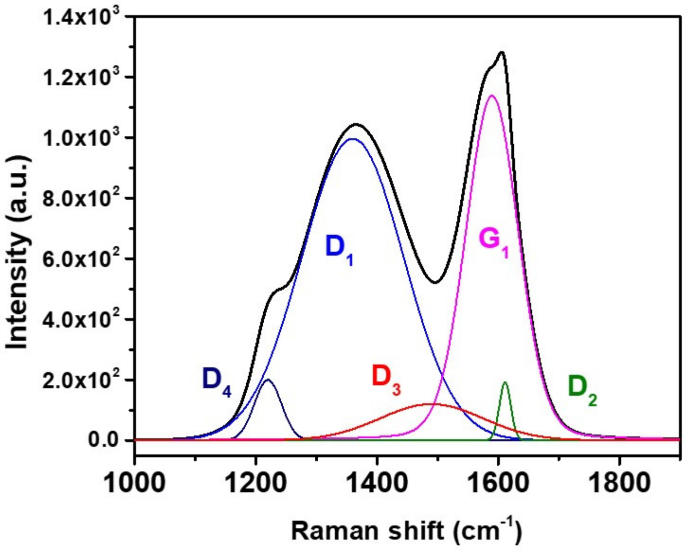

Figure S3: Raman spectra of Si@C NPs. These deconvolutions highlight the low degree of organisation of the C-shell. The detailed explanation of the various components and associated interpretations can be found elsewhere [66,67].

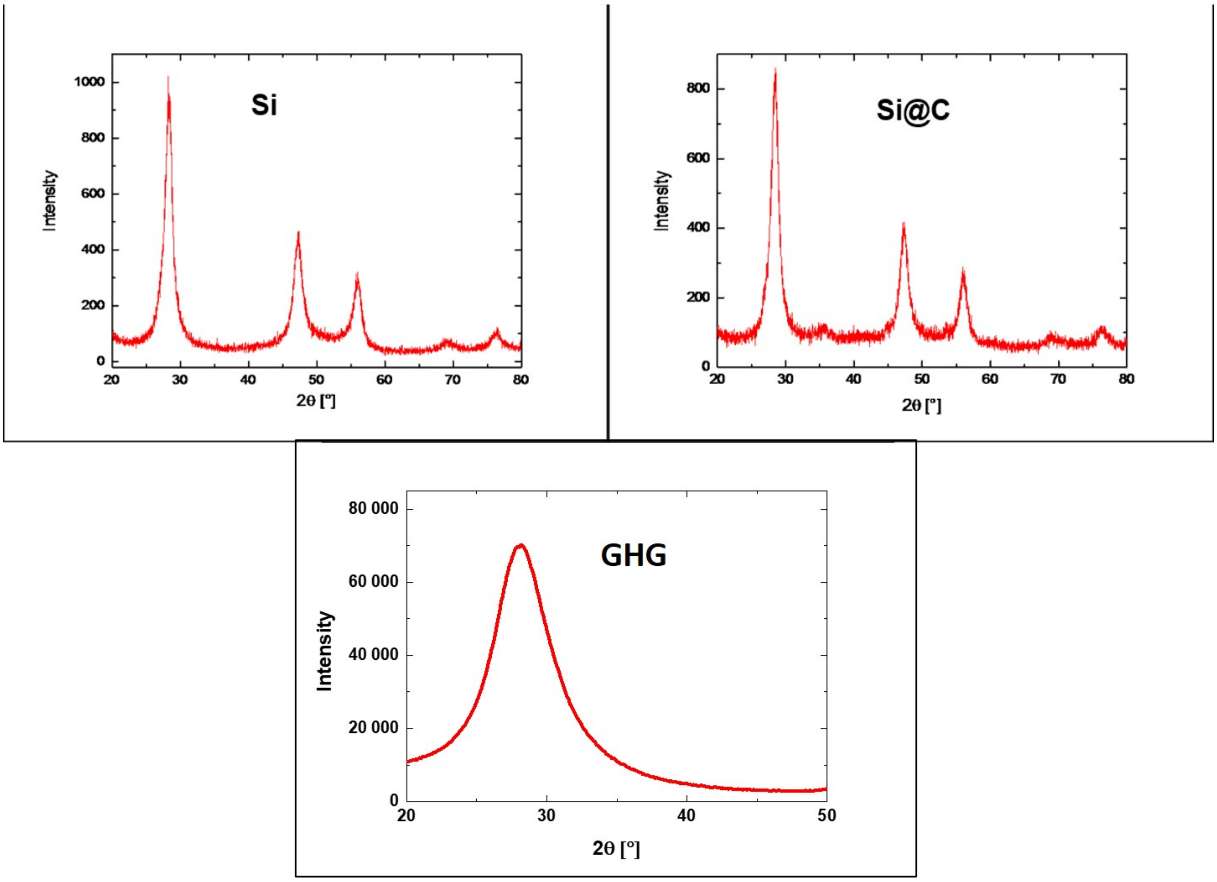

Figure S4: complementary XRD diffractogram of the sample references: Si, Si@C (Cu Kα radiation source) and GHG (Co Kα radiation source).

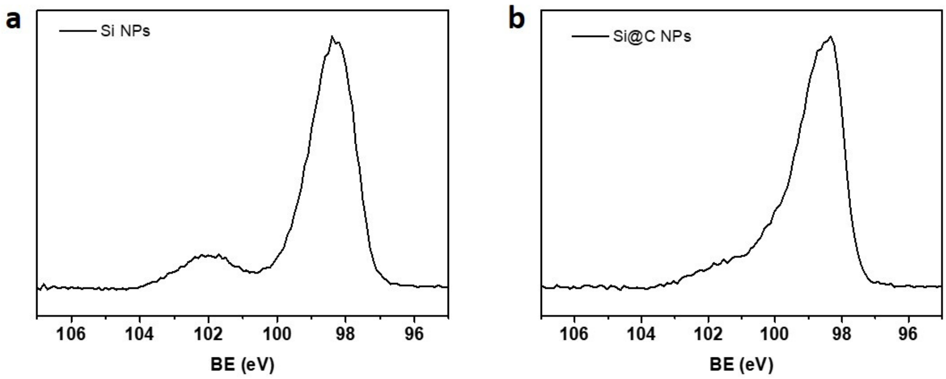

Figure S5: Si2p HR XPS spectra of pristine Si (**a**) and Si@C NP samples (**b**), showing the low intensity of the SiO_2 contribution.

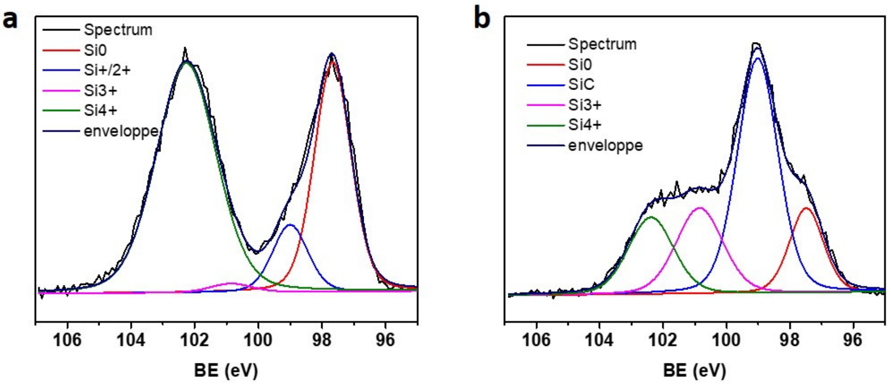

Figure S6: Deconvolutions of the HR Si2p XPS spectra recorded for Si-GHG (**a**) and Si@C-GHG (**b**) after 1h of reaction.

Table S1: Recapitulation of the deconvoluted Si2p components (peak position, FWHM values and atomic concentrations) attributed to Si0, SiC, Si+/Si^{2+}, Si^{3+} and Si^{4+}, along with ratios highlighting the stability of Si@C NPs-GHG composite compared to the more oxidation-sensitive Si-GHG composites (clear decrease of the Si^0/SiOx with increasing reaction time).

Attribution	Si^0		SiC		Si^+		$Si^{2+/3+}$		Si^{4+}		Si^0/SiO_x	Si^0+SiC/SiO_x	Si^0/SiC
	Peak position (eV) (FWHM)	%at	Peak position (eV) (FWHM)	%at	Peak position (eV) (FWHM)	%at	Peak position (eV) (FWHM)	%at	Peak position (eV) (FWHM)	%at			
**Si-GHG-1h**	97.67 1.36	5.41	-	-	99 1.30	1.52	100.86 1.52	0.23	102.27 2.22	8.85	0.5	-	-
**Si-GHG-18h**	97.13 1.40	1.51	-	-	98.85 1.30	0.17	101.17 1.52	3.37	102.28 2.22	7.87	0.1		
**Si@C-GHG-1h**	97.49 1.26	2.28	99.02 1.50	7.61	-	-	100.86 1.79	3.18	102.41 1.66	2.76	0.4	1.3	0.3
**Si@C-GHG-18h**	97.61 1.27	1.89	99.12 1.44	9.14	-	-	100.94 1.66	3.69	102.54 1.66	3.99	0.3	1.4	0.2
**[68]**	98.8	-	-	-	100	-	101.8	-	102.8	-	-	-	-

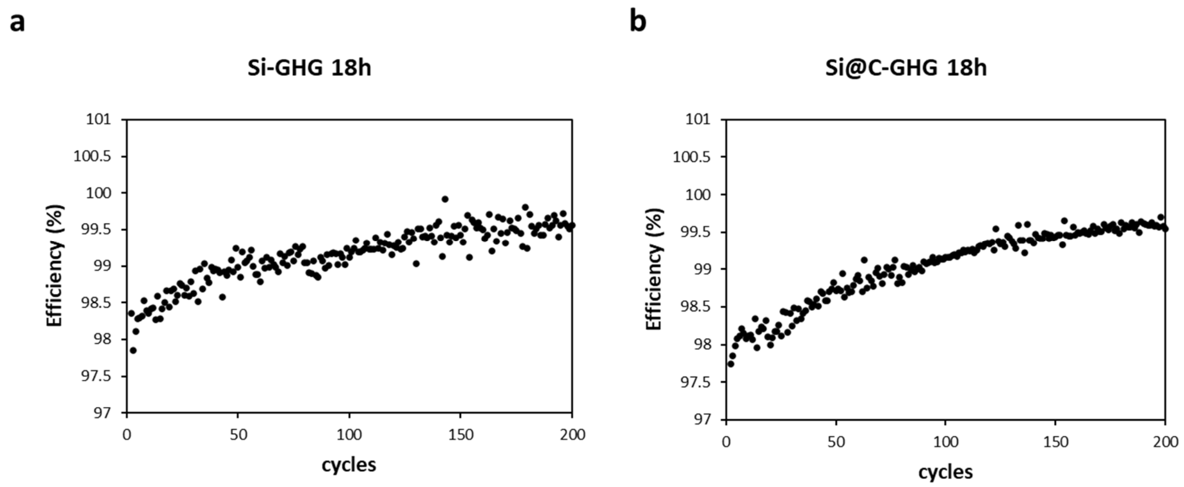

Figure S7: Coulombic efficiency of Si-GHG-18h (**a**) and Si@C-GHG-18h (**b**) during cycling.

Table S2: Comparison of different electrochemical performances obtained for electrode materials containing Si or Si@C NPs and graphene as an additive (in orange) or as a 3D scaffold (in green) described in the recent literature. It is noteworthy that the frontier between graphene as 2D or 3D component is not always clear-cut.

Sample designation	Sample description	Cell configuration	Electrolyte	Test parameters (formulation, mass loading, cell type, voltage range)	C_{SP} at first cycles	C_{SP} at xxx cycles	[Ref]
**C-Si/cGr**	carbonized Si NPs capped with glucose to form C-Si mixed with spray dried GO and ascorbic acid (50:50 in mass)	Half-cell	1 M $LiPF_6$ in EC, EMC, DMC	C-Si/cGr, Super P, CMC (80:10:10)<br>Loading 1,3 mg/cm^2 (full electrode material)<br>Coin cell CR2016<br>V window: 0-3V	1928 mAh/g intitial discharge capacity 0,5 C<br>1288 mAh/g at 0,1C<br>654 mAh/g at 2C	790 mAh/g after 100 cycles at 0,5C	**[39]**
**Si@NC/Graphene**	Si NPs mixed with graphene and egg white, calcinated under Ar<br>N-doped C shell around the SiNPs<br>Graphene (7% of Si in weight)		1 M $LiPF_6$ in DEC, EC,FEC<br>V window: 0,01-1,5V	Si@NC/Graphene, acetylene black, sodium alginate (6:2:2)<br>Loading 1,5-1,8 mg/cm^2<br>Coin-type cell	~1900 mAh/g (initial charge capacity)<br>1650 mAh/g (20th cycle at 0,1 A/g)	1230 mAh/g at 100th cycle	**[40]**
**C-Si@G**	APS coated SiNPs in GO matrix – freeze-dried mixed with PVDF (3:2) and carbonized under inert atm. – ratio of Si to GO 10:1?	Half-cell	1M$LiPF_6$ in EC, DMC	Loading 0,5 mg/cm^2<br>No extra binder as composite already carbonized with PVDF<br>V window 0,02-1 V	2523 mAh/g at 0,5A/g<br>752 mAh/g at 5 A/g	1987 mAh/g after 100 cycles	**[41]**
**Si/c-PEG/G**	Si mixed citric acid and PEG and GO followed by carbonization at 600°C under Ar	Full cell (cathode in LCO)<br>Pre-lithiation	1M $LiPF_6$ EC, DEC with 5% FEC	active material, PAA, Super P (70:15:15)<br>Cu foil<br>1 mg/cm^2 of active materials<br>Coin-cell	>1200 mAh/g from 0,2 to 1A/g	534 mAh/g after 100 cycles at 20 A/g	**[42]**
**C@Si/rGO**	GO mixed with PDDA@Si NPs – freeze-dried and thermally reduced at 500°C	Half-cell	1M $LiPF_6$ in EC/DEC with 5% FEC	full electrode weight (wafer, Si, C, rGO) 1,8 mg/cm^2<br>Coin-cell<br>V window: 0,01-1,5V	1988 mAh/g (1st discharge)	1155 mAh/g after 100 cycles	**[43]**
**Si@C/RGO**	PANI polymerization around Si NPs, mixed with GO and annealed at 600°C	Half-cell	1M $LiPF_6$ in EC, DEC	Active material, Super P, CMC-Na (70:25:5)<br>Loading mass 2 mg<br>Coin-type cell CR2016<br>V range: 0,01-1,5 V vs li/Li+<br>0,2 mV/s	2011 mAh/g (1st discharge at 0,3 A/g)	1121 mAh/g after 230 cycles at 0,3 A/g	**[45]**
**Graphite/Si/RGO (SGG)**	Mixture –self assembly followed by annealing – no 3D<br>No Si C coating – graphene is the	Full cell<br>Prelithiation in half cell	1M $LiPF_6$ in EC, DEC	Si 8%<br>2 mg of active material with different Si loading<br>cut-off at 2-3.8V<br>V range: 0.01-1.5V<br>with Super P and CMC-Na (70:15:15)	572 mAh/g at 0,2 C	502 at 0.8 C<br>After 600 cycles (95% retention)	**[46]**

**C@Si/GN/CNT/PDA-C**	Si NP coated with CTAB mixed with graphene and CNT, then dopamine; annealing at 600°C<br>Ratios of SiNPs, graphene, CNT (5:1:1) + PDA	Half-cell	1M $LiPF_6$ in EC, DEC	Active material, acetylene black, sodium alginate (8:1:1)<br>Loading: 1,3-1,5 mg/cm^2<br>Coin-type cell 2025<br>V window: 0,01-1,5 V	2431 mAh/g (1st discharge caapcity at 0,1 mV/s	1946 mAh/g at 0,1 A/g after 100 cycles<br>1306 mAh/g at 1A/g after 100 cycles	**[55]**
**G-3DGNs-Si**	MCMB oxidation/expansion to 3DGNs, Si NPs incorporation and annealing (50:50)<br>- No C Shell around the Si NPs	Half-cell	1M $LiPF_6$ in EC, ethyl carbonate, FEC	G-3DGNs-Si, sodium alginate, Super P (70, 20, 10<br>CR2025<br>V window 0,01-2,5 V	1806,8 mAh/g initial discharge at 0,2 A/g<br>890 mAg/g at 5 A/g	817,3 mAh/g at 1 A/g after 150 cycles	**[56]**
**PANI/Si@RGO**	PANI coated on SiNPs mixed with GO alcogel (2:1 in mass) and further subjected to hydrothermal process to produce an aerogel that was pyrolyzed	Half-cell	$LiPF_6$ in EC, DEC, VC	PANI/Su@RGO, Super P and PVDF (80:10:10)<br>Coin cell<br>Full mass loading of 0,5 mg	1844 mAh/g at 0,2 A/g<br>1180 mAh/g at 1 A/g<br>3036 at 0,1 A/g	880 mAh/g after 200 cycles at 1 A/g	**[57]**
**Si-G-x**	PVP, Si NPs, GO mixed, and hydrothermal formation of hydrogel – followed by different drying time<br>Ratio GO:Si NPs 2:1<br>No C shell on the Si NPs	Half-cell	1M $LiPF_6$ in EC, DEC	Si/RGO composite, Super P, CMC + styrene butadiene (60:20:20)<br>Loading mass ~0,7 -1,65 mg/cm^2<br>Coin-type cell CR2032<br>V range: 0,005 – 2V<br>0,1 mV/s	1563 mAh/g (initial charge capacity at 0,05A/g)	1210 mA h g1 after 100 cycles at 0.5 A g1	**[58]**
**Si@PDA@rGO hydrogel or SCG**	DA polymerization/annealing around Si NPs mixed with GO, ascorbic acid and formation of the aerogel at 80°C for 8h and annealed at 700°C<br>3D graphene structure<br>Si with a PDA N-doped C shell<br>Different Si:G ratios	Half-cell	1M $LiPF_6$ in DMC, EMC, EC	SCG, Super P, PVDF (8:1:1)<br>Loading of 0,8-1 mg/cm^2 (electrode material)<br>Coin-type cell CR2025<br>V window: 0,01-3V	1618,1 mAh/g (1st discharge capacity – scan rate: 0,1 mV/s)	712 mAh/g at 100 mA/g after 100 cycles<br>402 mAh/g at 1 A/g after 1500 cycles	**[61]**
**Si@C-GHG**	Si NPs coated with C shell obtained from laser pyrolysis, mixed with GO, hydrothermal process to get the aerogel structure<br>Ratio Si:graphene: 50:50	Half-cell	1 M $LiPF_6$ in ED, DEC with FEC and VC	Electrode material, CMC (8:2)<br>No additional conductive additive<br>Loading 0,8 mg/cm^2<br>Results express per Si active material ie. 0,3-0,4 mg/cm^2<br>Coin-type cell	2500 mAh/g (initial capacity at C/5)<br>1250 mAh/g at 2C	2205 mAh/g after 200 cycles at C/5	**This work**
